# Classifying zones of suitability for manual drilling using textural and hydraulic parameters of shallow aquifers: a case study in northwestern Senegal

**DOI:** 10.1007/s10040-017-1642-9

**Published:** 2017-08-14

**Authors:** F. Fabio Fussi, Letizia Fumagalli, Francesco Fava, Biagio Di Mauro, Cheik Hamidou Kane, Magatte Niang, Souleye Wade, Barry Hamidou, Roberto Colombo, Tullia Bonomi

**Affiliations:** 1 Department of Environmental Science, University Milano Bicocca, Piazza della Scienza 1, Milan, Italy; 2 University of Thies, Thies, Senegal; 3 University Cheik Anta Diop, Dakar, Senegal; 4 SNAPE (Service Nationale de Points d’Eau), Conakry, Guinea

**Keywords:** Groundwater development, Unconsolidated sediments, Water supply, Drilling, Senegal

## Abstract

A method is proposed that uses analysis of borehole stratigraphic logs for the characterization of shallow aquifers and for the assessment of areas suitable for manual drilling. The model is based on available borehole-log parameters: depth to hard rock, depth to water, thickness of laterite and hydraulic transmissivity of the shallow aquifer. The model is applied to a study area in northwestern Senegal. A dataset of boreholes logs has been processed using a software package (TANGAFRIC) developed during the research. After a manual procedure to assign a standard category describing the lithological characteristics, the next step is the automated extraction of different textural parameters and the estimation of hydraulic conductivity using reference values available in the literature. The hydraulic conductivity values estimated from stratigraphic data have been partially validated, by comparing them with measured values from a series of pumping tests carried out in large-diameter wells. The results show that this method is able to produce a reliable interpretation of the shallow hydrogeological context using information generally available in the region. The research contributes to improving the identification of areas where conditions are suitable for manual drilling. This is achieved by applying the described method, based on a structured and semi-quantitative approach, to classify the zones of suitability for given manual drilling techniques using data available in most African countries. Ultimately, this work will support proposed international programs aimed at promoting low-cost water supply in Africa and enhancing access to safe drinking water for the population.

## Résumé

Une méthode est. proposée utilisant l’analyse des logs stratigraphiques des forages pour la caractérisation des aquifères peu profonds et pour l’évaluation des zones favourable pour le forage manuel. Le modèle repose sur l’analyse des paramètres extraits des logs de forage disponibles: profondeur au socle, profondeur au niveau piézométrique, épaisseur de la latérite et transmissivité hydraulique de l’aquifère peu profond. Le modèle est. appliqué à une zone d’étude dans le nord-ouest du Sénégal. Un ensemble de données de logs de forages a été traité en utilisant l’application informatique TANGAFRIC développée au cours de la recherche. Après une procédure manuelle pour attribuer une catégorie standardisée décrivant les caractéristiques lithologiques, l’étape suivante et une extraction automatique des différents paramètres texturaux et l’estimation de la conductivité hydraulique en utilisant des valeurs de référence disponibles dans la littérature. Les valeurs de conductivité hydraulique estimées à partir des données stratigraphiques ont été partiellement validées, en les comparant aux valeurs mesurées à partir d’une série de pompages d’essais réalisés sur des forages de grand diamètre. Les résultats indiquent que cette méthode permet de produire une interprétation fiable du contexte hydrogéologique peu profond en utilisant des informations généralement disponibles dans la région. La recherche contribue à améliorer l’identification de régions pour lesquelles des conditions sont en faveur de forages manuels. Ceci est. réalisé en appliquant la méthode décrite, basée sur une approche structurée et semi-quantitative, pour classer l’aptitude de zones à des techniques données de forage manuel en utilisant les données disponibles dans la plupart des pays africains. En fin de compte, ce travail appuiera les programmes internationaux proposés visant à promouvoir l’approvisionnement en eau potable à un faible coût en Afrique et à améliorer l’accès à l’eau potable pour la population.

## Resumen

Se propone un método que utiliza el análisis de registros estratigráficos de pozos para la caracterización de acuíferos poco profundos y para la evaluación de áreas aptas para la perforación manual. El modelo se basa en el análisis de parámetros extraídos de registros de pozos disponibles: profundidad de la roca dura, profundidad del agua, espesor de laterita y transmisividad hidráulica del acuífero poco profundo. El modelo se aplica a un área de estudio en el noroeste de Senegal. Un conjunto de datos de registros de perforaciones se ha procesado utilizando un paquete de software (TANGAFRIC) desarrollado durante la investigación. Después de un procedimiento manual para asignar una categoría estándar que describe las características litológicas, el siguiente paso es la extracción automatizada de diferentes parámetros texturales y la estimación de la conductividad hidráulica usando los valores de referencia disponibles en la literatura. Los valores de conductividad hidráulica estimados a partir de datos estratigráficos han sido parcialmente validados, comparándolos con valores medidos de una serie de ensayos de bombeo realizados en pozos de gran diámetro. Los resultados muestran que este método es capaz de producir una interpretación confiable del contexto hidrogeológico superficial usando información generalmente disponible en la región. La investigación contribuye a mejorar la identificación de áreas donde las condiciones son aptas para la perforación manual. Esto se logra aplicando el método descrito, basado en un enfoque estructurado y semicuantitativo, para clasificar la idoneidad para determinadas técnicas de perforación manual utilizando datos disponibles en la mayoría de los países africanos. En última instancia, este trabajo apoyará los programas internacionales propuestos para promover el suministro de agua a bajo costo en África y mejorar el acceso al agua potable para la población.

## 摘要

提出了一种利用井孔地层测井资料分析浅层含水层的分析方法,并对适合人工钻孔的区域进行评估。该模型基于从可用井眼测井中提取的参数分析:浅层含水层深度与硬岩,水深,红土厚度和液压透过率。该模型适用于塞内加尔西北部的一个研究区域。钻孔日志的数据集已经使用研究期间开发的软件包(TANGAFRIC)进行处理。在指定描述岩性特征的标准类别的手动程序之后,下一步是使用文献中可用的参考值自动提取不同的结构参数和水力传导率的估计。通过与大直径井进行的一系列泵送试验的测量值进行比较,已经部分验证了从地层数据估算出的水力传导率值。结果表明,该方法能够利用该地区通用的信息,对浅层水文地质环境产生可靠的解释。该研究有助于改善条件适合手动钻孔的区域的识别。这是通过应用所描述的方法,基于结构化和半定量方法,使用大多数非洲国家可用的数据对给定的手动钻孔技术进行分类。最终,这项工作将支持旨在促进非洲低成本供水和提高人民获得安全饮用水的拟议国际方案。

## Resumo

Um método é proposto usando análise estratigráfica de perfis de poços para a caracterização de aquíferos rasos e avaliação de áreas favoráveis para perfuração manual. O modelo é baseado na análise dos parâmetros extraídos de perfis de poços disponíveis: profundidade da rocha dura, profundidade da água, espessura da laterita, e transmissividade hidráulica do aquífero raso. O modelo é aplicado a uma área de estudo no noroeste do Senegal. Os perfis dos poços foram processados usando o programa de computador (TANGAFRIC) desenvolvido durante a pesquisa. Após um procedimento manual para assinalar uma categoria padrão descrevendo as características litológicas, o próximo passo é a extração automática de diferentes parâmetros texturais e a estimativa da condutividade hidráulica usando os valores de referência disponíveis na literatura. Os valores de condutividade hidráulica estimados de dados de estratigrafia foram parcialmente validados comparando estes com valores medidos através de uma série de testes de bombeamento realizados em poços de grande diâmetro. Os resultados mostram que este método é capaz de produzir uma interpretação confiável do contexto hidrogeológico raso usando informações disponíveis na região. A pesquisa contribui para promover a identificação de áreas onde as condições são favoráveis para perfuração manual. Isso é obtido aplicando o método descrito, baseado no processo estruturado e semiquantitativo para classificar a favorabilidade para uma certa técnica de perfuração manual usando os dados disponíveis na maioria dos países Africanos. Por último, este trabalho suportará programas internacionais propostos com o objetivo de promover o abastecimento de água a baixo custo na África e aumentar o acesso seguro a água potável para a população.

## Introduction

Despite overall progress, 748 million people still had no access to safe drinking water in 2012, 325 million (43%) of whom were living in Sub-Saharan Africa (WHO/UNICEF 2014). Groundwater has proven to be the most reliable resource for meeting rural water demand in Sub-Saharan Africa, and it can derive from different types of aquifers. Unconsolidated sediments cover 22% of Sub-Saharan Africa, and at least 60 million people in rural areas obtain water from this type of aquifer (MacDonald and Davies 2000).

Manual drilling refers to several drilling methods that rely on human energy to construct a borehole (i.e. a narrow hole bored in the ground using some kind of drilling tools) and complete a water supply (Danert 2015). These techniques are well established in certain countries, e.g. Bangladesh, Bolivia, India, Kenya, Niger, Nigeria and Madagascar. In recent years, however, there has been an increased interest in promoting manual drilling in regions where this technique is rarely applied even though it could improve access to clean water. UNICEF (United Nations International Children’s Emergency Fund) has been involved in this process in Africa since 2004 (Gaya 2004). Other organizations have gathered experience in pilot projects to promote manual drilling in different regions (e.g. Forsyth et al. 2010); these techniques are now becoming increasingly relevant in several countries (Danert 2009, 2015).

Although different techniques for manual drilling are available (Carter 2005; Danert 2015), they can be applied only where shallow geological layers are relatively soft (i.e. it is possible to drill using manually powered tools) and the water table is not too deep (i.e. that exploitable water strikes occur within a depth range achievable with manual drilling techniques; 50 m can be a realistic value for the maximum depth commonly reached by hand-drilled wells, although there are examples that reach up to 100 m). Therefore, the promotion of manual drilling to improve water supply requires a preliminary identification of those zones with suitable hydrogeological conditions. Such an approach was initially tested in Chad using the water points’ database of the national water authorities (Direction de l’Hydraulique) and a simple procedure of visual interpretation of the variations in static water level and the distribution of geological units (Gaya et al. 2005). The Chad study was used for an intense program of promotion of manual drilling by UNICEF and the national water authority, which resulted in thousands of new excellent hand-drilled wells in the country (Danert 2015), with a network of private contractors trained in high-quality construction techniques. Following the Chad experience, UNICEF produced maps of suitable zones at country level within Africa, applying a schematic model with some modifications in 12 countries between 2008 and 2010 (Fussi 2011; Fussi et al. 2013). Suitable zones for manual drilling were derived from the analysis of three main parameters: geological suitability (depending on the thickness and permeability of shallow layers), water depth suitability (depending on static water level in the first aquifer) and morphological suitability (based on the existence of landforms that facilitate the accumulation of unconsolidated sediments or erosion). This method was based on the analysis of water point databases, geological maps and the Shuttle Radar Topography Mission (SRTM) digital elevation model, integrated with qualitative experience of local hydrogeologists. Major relevance is given to the national water point database, i.e. the centralized archive of water point data generally held by the national water authority in each country. This source of information contains a large amount of data but generally presents several problems in their quality (not up-to-date, duplicated records, incorrect and incomplete information). These data were analysed only through a simple qualitative visual interpretation (a robust and quantitative procedure of elaboration was not proposed). A similar approach derived from a cross analysis of shallow geology (obtained from the simplification of geological maps) and depth to water (assigning an estimated range of this parameter to the category of landforms obtained by processing the SRTM digital elevation model) was used in Madagascar (Salama 2008). A different method was applied in Tigray, northern Ethiopia (Huisman Foundation 2014). This approach is based on direct field survey (observation of landscape characteristics, soil type and water level in hand-dug wells) and discussion with local experts.

A method to investigate one of the relevant aspects to consider for the feasibility of manual drilling (water depth) was proposed in Niger (Thomas et al. 2012). It is based on a multivariate statistical procedure for the estimation of water depth through an indirect set of information (remote sensing, terrain modelling, thematic maps). Even though it does not propose a complete method for the identification of suitable zones, this research does suggest a method to characterize the shallow hydrogeological context.

Regardless of the sources of information, all these methods to determine the suitability of an area for manual drilling take the same criteria into consideration: properties of the shallow geological layers and water-level depth. The study in Tigray had a detailed field survey and therefore is applicable only for relatively small study areas; however, the other methods all have similar critical aspects:

- They depend on existing information (like thematic maps, qualitative experience of local experts) that is often incomplete and does not homogeneously cover the whole country, leading to unreliable interpretation where this information is limited.
- They can support regional- and national-scale analysis, but they are not applicable at local level, given the coarse scale of the mapping products and low spatial density of information.
- They are largely based on qualitative perception and assessment of experts, without a structured data analysis procedure. This makes it difficult to replicate the study and compare the results in different areas.

The main objective of this study is to propose a new method to identify suitable zones for manual drilling, based on a semi-quantitative estimation of hydrogeological data and a robust process of data analysis. Specifically, the proposed approach aims at improving existing methods by:

- A more structured and quantitative procedure to analyse existing geological datasets, especially those coming from national water point databases and borehole stratigraphic logs.
- A systematic approach for borehole data processing, with inclusion of information available only in hard copy and a limited field survey for a general validation. Integrating this information makes it easier to increase the level of detail of the mapping products, compared with previous maps at regional or country level.
- A more objective, systematic and replicable workflow.

## Materials and methods

### Description of the study area

**Fig. 1:**
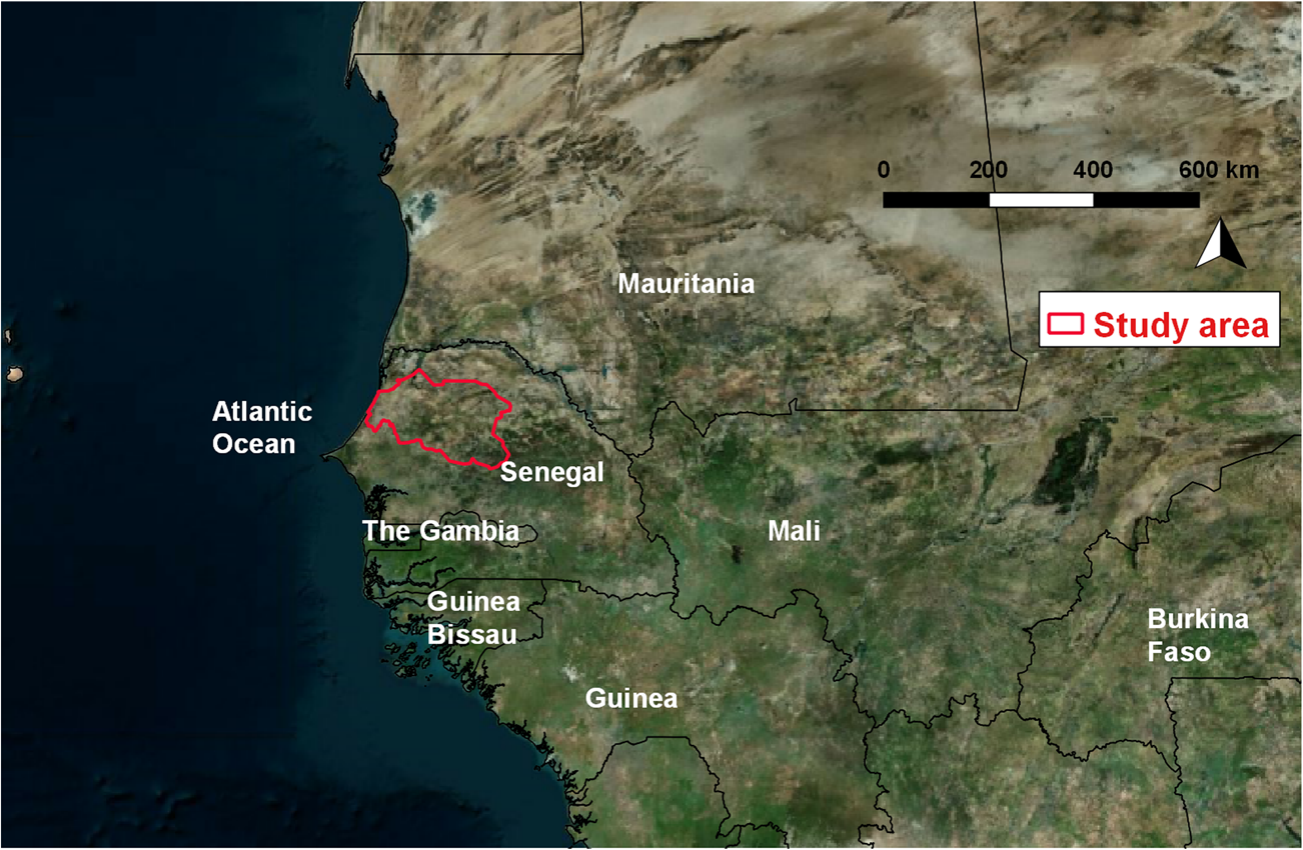
Location of the study area, the Louga region (delineated by a *red line*) in Senegal

The study area is the Louga region in north-western Senegal, between 14°70′ and 16°10′ north and 14°27′ to 16°50′ west (Fig. 1), and covers 24,874 km^2^. It was selected in order to target zones with different shallow hydrogeological features and different suitability for manual drilling, as obtained from the existing study in the framework of the UNICEF program (Kane et al. 2013).

The total population of the study area is 880,482 inhabitants (more than 500,000 inhabitants in rural areas), with 57% having access to safe drinking water and 17% having adequate sanitation (PEPAM 2015). Climatic conditions are characteristic of the Sahelian regime, with rainfall approximately 350–500 mm/year (with a decreasing trend moving to the north), concentrated between June and October. Along the coastal area, there is the effect of a humid wind brought by the Azores Anticyclone. Morphology is mainly flat, with limited undulation formed by sandy dunes. From the geological point of view, the study area is situated in the Tertiary Senegalo-Mauritanian sedimentary basin, constituted by interbedded layers of limestone, sandstone, marl and clay, and elongated in the N–S direction for 1,400 km from Mauritania to Guinea Bissau. This sedimentary basement is covered by sands or sandy clay. Moving eastward, there are different sandy formations (with an increase in the presence of clay): the coastal dunes, the Ogolian red dunes, and the Continental terminal.

### Source of data

The method is based mainly on a detailed analysis of previously existing data, most of it acquired free of charge. Direct field data collection is limited and principally focused to validate the interpretation. The main sources of data are thematic maps and hydrogeological information obtained from the national database of water points of Senegal. Geology, soils, morphopedology and land-cover datasets have been obtained from 1:500,000 maps published for the national plan for land-use and development (The Remote Sensing Institute South Dakota State University 1986). Water points data were obtained from the national inventory held by DGPRE (Direction de la Gestion et de Planification des Ressources en Eaux) in Senegal; this database is part of the geographic information system of water resources (Ré﻿publique du Sénégal 2000) from SGPRE (Service de Gestion et de Planification des Ressources en Eau), and it is managed with the software PROGRES (ANTEA/BURGEAP 2007). Three categories of data have been obtained from the water point database:

1. General inventory of water points (1,277 records in the study area, including deep boreholes and hand-dug wells). They cover all the study area, although there is a much higher concentration on the western side. From this dataset, it was possible to obtain the position and total depth of the boreholes/wells and the depth to static water level.
2. Inventory of piezometers (45 records in the study area, almost completely concentrated in the coastal region, not more than 50 km away from the sea). The piezometers have the same information as the general inventory of water points, plus the description of the main aquifer and a few time-series of static water level observations.
3. Stratigraphic logs of boreholes (131 in the study area, mainly concentrated on the western side). These logs have the same information as the general inventory, plus the lithological description and position of different layers found during the drilling. No stratigraphic logs are available for hand-dug wells.

Since the information of elevation stored in the national water point database proved to be unreliable, it was neglected, and the elevation was obtained from publicly available digital terrain models (Aster Global Digital Elevation Model v.2, 30-m resolution). The national water point database stores information concerning mechanized boreholes, but limited attention is paid to large-diameter shallow wells. Furthermore, stratigraphic logs are generally not detailed for shallow layers, and data about hydraulic parameters refer to deep, fractured aquifers. Concerning the use of static water-level data to estimate the depth to the shallow water table, two aspects must be carefully considered:

1. Most of the data (from boreholes and piezometers) refer to a deep fractured aquifer, whose piezometric level can differ from the shallow water table in the case of confining layers between the two aquifers (as in the central-eastern side of the study area); here, the estimation of the depth of water that can be exploited using manual drilling is uncertain.
2. Static water level in the national water point inventory was measured in different years and seasonal conditions. The difference in water level could be the consequence of temporal changes and may not be related to the piezometric gradient of the water table. However, the extremely limited information available from the temporal series of static water level (data from four piezometers in the coastal zone, whose water level was measured in October 2011 and August 2014) shows a change over time smaller than 30 cm.

### Classification of zones of suitability for manual drilling

The methodology proposed in this report to classify suitable zones for manual drilling is based on a structured and semi-quantitative analysis of available borehole log data. The method can be applied in three steps: (1) assessment of feasibility, (2) estimation of potential for exploitation, (3) final classification of suitability.

#### Assessment of feasibility

**Fig. 2:**
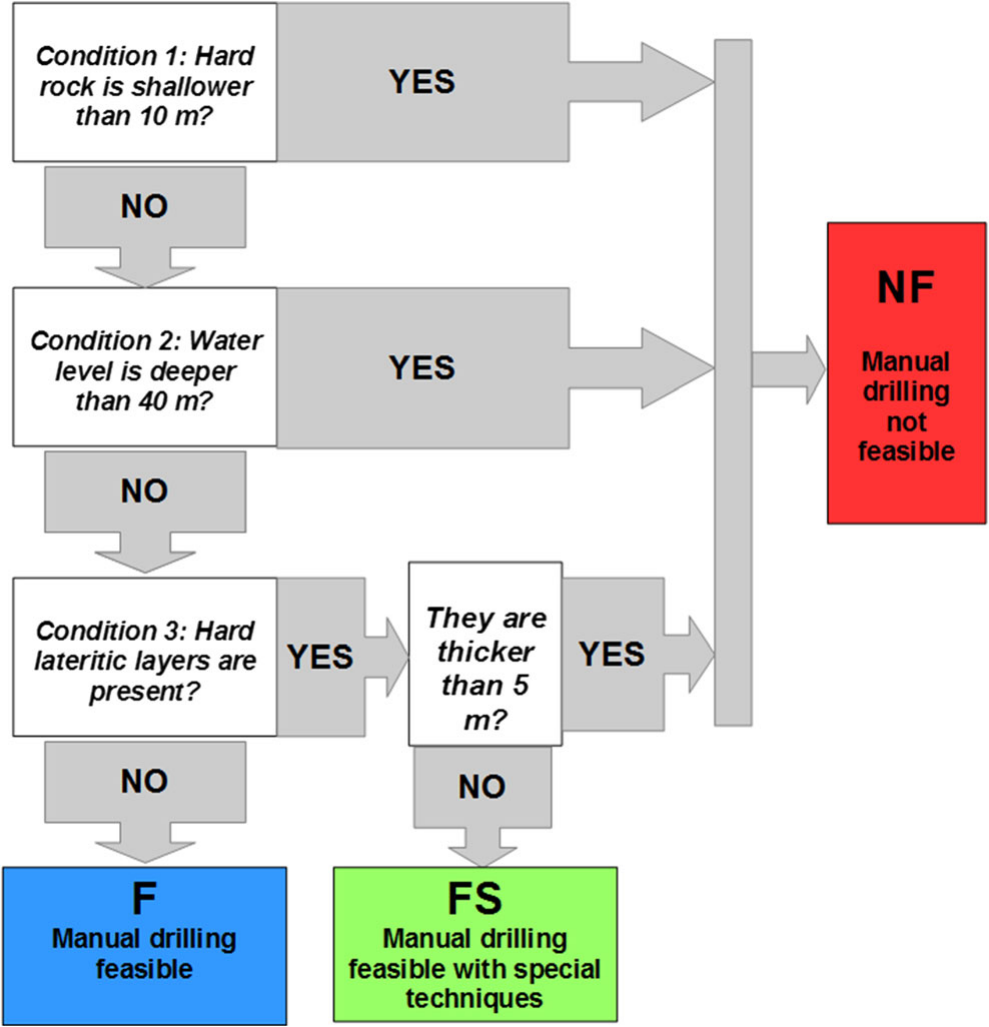
Schematization of the procedure to assess feasibility of manual drilling. *F* feasible, *FS* feasible with special techniques, *NF* not feasible

Assessing the feasibility of manual drilling in a specific location means evaluating whether the existing hydrogeological conditions allow the completion of a hand-drilled well (with the different techniques available). This assessment was carried out by analysing two main parameters extracted from borehole logs: the presence of hard layers and the depth to the water level. The locations of hard stratigraphic layers can be derived from the locations of hard rock (in this case the layers generally represent the maximum possible depth of manual drilling in that area) or compact laterite (the layers are intercalated between unconsolidated sediments, and they can be broken with special manual drilling techniques, if they have limited thickness, and drilling can continue deeper). Based on these considerations, the procedures to assess feasibility for manual drilling can be schematized as a sequence of three conditions, evaluated through Boolean operators (yes/no), and schematized in Fig. 2:

- *Condition 1: depth to hard rock*. In the assessment of feasibility for manual drilling, hard rock is considered a solid layer which has significant hardness and compactness, and which is generally impossible to perforate using drilling tools operated by human energy (drilled without power obtained from mechanized machine). In terms of the geological process, such hard rock layers could correspond to the bottom of the weathered and unconsolidated material covering the fresh compact rock, or the lower limit of unconsolidated layers derived from the depositional process on top of the basement rock. The presence of hard rock at the ground surface, or shallower than 10 m, means that manual drilling is not recommendable; even in the case of a water table close to the ground surface, a water-column depth of maximum 10 m leads to unreliable water supply from the wells, especially during dry periods (when the water table becomes deeper). Therefore, in this situation, a complete and successful hand-drilled well is considered not feasible.
- *Condition 2: depth to water*. This parameter refers to the minimum depth of water strikes that can be attained in hand-drilled wells. In the case of the unconfined water table, the piezometric level corresponds to the first water strike, while in the case of confined aquifers, there are different levels. As mentioned in section ‘Introduction’, a reference value of 50 m was assumed as the maximum depth commonly achievable using manual drilling; this seems to be consistent with previous experience in Senegal. Given this assumption, and considering that a few metres of water column is needed to ensure a positive result, the limit of 40 m as a maximum water-level depth was kept as a threshold for the feasibility of manual drilling.
- *Condition 3: presence of hard laterite*. One common weathering product in West African soils is laterite, which is rich in aluminium and is sometimes present in very hard layers and in some other situations as a reddish, clay-rich, unconsolidated material. Hard lateritic crusts can be perforated if their thickness is limited, but special techniques (e.g. percussion) are required. Based on direct experience of manual drilling experts, perforation is probably possible for a hard lateritic crust thinner than 5 m, while if the layers are thicker, manual drilling is considered not possible.

With this procedure, three classes of *feasibility* can be distinguished (Table 1): not feasible (NF), feasible (F) and feasible with special techniques (FS).

**Table 1:** Classes of feasibility for manual drilling

Class of feasibility	Description
Not feasible (NF)	Not feasible because of the presence of shallow hard rock, large depth to water, and a thick lateritic hard layer
Feasible (F)	Manual drilling can be successfully done in this hydrogeological context
Feasible with special techniques (FS)	Manual drilling can be done, but the presence of hard intercalated layers necessitates the use of special techniques at a certain depth (e.g. percussion) to break it, together with other common methods to drill in unconsolidated sediments

#### Classification of the potential for exploitation

After having identified where manual drilling is feasible (i.e. classes F and FS, with the option of special techniques required), the second step is the classification of the *potential for exploitation* in these zones. This classification can give an indication of the expected yield, availability of water during the dry season (because of seasonal fluctuation of the water level), the type of pump that can be installed and the size of the population served. The potential yield of the well is related to hydrogeological factors (i.e. the geometry and hydraulic characteristics of the target aquifer) and engineering aspects (quality of construction and performance of pumping system). In the proposed methodology, the hydrogeological aspects have been classified by extracting two parameters from the analysis of borehole logs with the procedure described in section ‘Data processing and interpretation: thickness and hydraulic conductivity (*K*) of saturated layer. The potential for exploitation with manual drilling is related to the hydraulic transmissivity in the exploitable interval (*T*_ex_), which itself is calculated as shown in Eq. (1)

1 $$ {T}_{\mathrm{ex}}={K}_{\mathrm{ex}}\times {H}_{\mathrm{ex}} $$

where *T*_ex_ is the hydraulic transmissivity (m^2^/s) of the exploitable layer by manual drilling (this means up to 50 m deep). *H*_ex_ is total thickness (m) of the saturated exploitable layers, corresponding to the difference between static water level and 50 m, if the upper limit of hard-rock-layer depth is >50 m or the difference between static water level and the upper limit of the hard-rock layers if <50 m. *K*_ex_ is average hydraulic conductivity (m/s) in the saturated exploitable layer.

The potential for exploitation is considered in relation to the performance of different pumping systems available for hand-drilled wells (Table 2). Five classes of potential for exploitation are defined (Table 3). This generic approach can be applied to different regions although threshold values of *T*_ex_ are related to site-specific conditions (e.g. local characteristics of aquifers, depth of water table, seasonal fluctuations).

**Table 2:** Types of pumps usually installed on hand-drilled wells and their expected yield

Type of pump	Description and usage
Hand pump	The most common pumping system for hand-drilled wells. Although the aquifer could be more productive, the expected yield from hand-operated pumps is approximately 0.2 L/s, with a maximum limit of 0.4 L/s in optimal conditions. They are suitable for the whole range of static water-level depth considered as feasible for manual drilling (maximum depth of static water level = 40 m) and they can provide water for groups of approximately 250 users for their domestic needs
Solar pump	Solar-powered pumps can be installed in highly productive hand-drilled wells. They can supply water for a larger community (and eventually connect to small water distribution systems). Their yield depends on the power generated by solar panels (whose extension and power production can vary considerably); as a reference value, 1 L/s is considered as a reasonable yield achievable with a solar pump in a productive aquifer, providing water to 1,000 users
Rope pump	A rope pump consists of a loose hanging rope that is lowered into a well and drawn up through a pipe that reaches the water. On the rope, round disks or knots matching the diameter of the pipe are attached which pull the water to the surface. These pumps are suitable and cheap systems to collect water from hand-drilled wells in the case of small communities (approximately 50 users). They have a low yield (0.1 L/s as reference value), and they can be installed only where the static water level is not deeper than 15 m

**Table 3:** Classes of potential for exploitation

Class of potential	Description
NP: potential null	Physically, manual drilling can be done, but the well will be dry, since the water level is deeper than the hard-rock depth (i.e. the maximum depth achievable by manual drilling). Therefore, the porous aquifer is completely dry (*H*_ex_ = 0)
LP: low potential	Hand-drilled wells can be equipped with hand pumps, but expected yield is low (less than 0.2 L/s). After intense pumping, or in the case of a decreasing water level, the well is likely to become dry
MP: moderate potential	Hand-drilled wells can be equipped with hand pumps and provide a reliable water supply; pumping cannot be continuous for a long time (for example, for 2 h or more, as is frequent during peak hours for water collection in rural areas in Africa). Not suitable for intense utilization by large groups (i.e. less than 100 users)
GP: good potential	Hand-drilled wells can provide a continuous water supply, with adequate yield using hand pumps. Suitable for medium-sized communities (approximately 250 users, which is often a guideline value of maximum users for hand pumps in water supply programs)
EP: excellent potential	Hand-drilled wells can provide an excellent yield (higher than 0.5 L/s). The well can supply water for a continuous utilization of hand pumps and can sometimes be equipped with solar pumps (providing water to a larger population)

#### Assigning the final class of suitability

The final class of *suitability* derives from the combination of feasibility and potential for exploitation. With this method, 11 possible combinations (Table 4) are defined, with three classes of suitability: not suitable, suitable with poor results, suitable.

**Table 4:** Final classification of suitability for manual drilling

Feasibility	Potential for exploitation	Combination	Class of suitability
Not feasible			
NF	–	NF	Not suitable
Feasible			
F	NP	F-NP	Not suitable
	LP	F-LP	Suitable with poor results
	MP	F-MP	Suitable
	GP	F-GP	Suitable
	EP	F-EP	Suitable
Feasible with special techniques			
FS	NP	FS-NP	Not suitable
	LP	FS-LP	Suitable with poor results
	MP	FS-MP	Suitable
	GP	FS-GP	Suitable
	EP	FS-EP	Suitable

*NP* potential null; *LP* low potential; *MP* moderate potential; *GP* good potential; *EP* excellent potential; *F* feasible; *FS* feasible with special techniques; *NF* not feasible

### Data processing and interpretation

Borehole-log data were processed with the software TANGAFRIC (Fussi et al. 2014), specifically designed for this purpose during this research. Four steps were followed:

1. Standardization and identification of common categories (on the basis of the most common stratigraphic terms in the datasets from Guinea and Senegal).
2. Assignment of standard categories to the description of each layer of the stratigraphic logs by means of manual codification by two local hydrogeologists, adapting the procedures used in the software TANGRAM at the University Milano Bicocca, Italy (Bonomi 2009; Bonomi et al. 2014).
3. Extraction of *textural composition of layers* from the interpretation of the codes corresponding to the main texture component, secondary texture component, and texture adjective.
4. Classification of the possible textural categories allowed in the coding process in five classes (Table 5): three classes discriminated on the basis of grain size (coarse, medium and fine) for unconsolidated sediments, and two other classes for hard layers (hard rock and hard laterite). Textural classification of sediments is derived from qualitative descriptions by drillers. However, a possible indication of the dimensions of particles for each category can be obtained from literature (e.g. Fetter 1994).

**Table 5:** Texture classes

Texture class	Categories
Coarse texture	Sand, gravel
Medium texture	Marl, silt
Fine texture	Clay, vegetal layer, lignite, laterite, alterite
Hard rock	Basalt, limestone, dolerite, gabbro, granite, sandstone, quartz, schist, silex
Hard laterite	Lateritic crust

The stratigraphic data were processed, and the percentage of each texture class (using a weighted average procedure) was extracted for a sequence of intervals with a regular step. Table 6 shows an example of a log with “sand” between depths 0 and 4 m and “sandy clay” between depths 4 and 10 m.

**Table 6:** Output table of TANGAFRIC with the distribution of texture classes for each 2-m interval

Input table		Output table after data processing					
Depth (m)	Texture	From (m)	To (m)	Coarse (%)	Medium (%)	Fine (%)	Hard (%)
From 0 to 4	Sand	0	2	100	0	0	0
		2	4	100	0	0	0
From 4 to 10	Sandy clay	4	6	30	0	70	0
		6	8	30	0	70	0
		8	10	30	0	70	0

Hydraulic conductivity (*K*) depends on texture (in the case of unconsolidated materials) or characteristics of fracturing (in the case of rocks). Ideally, the hydraulic conductivity of geological layers must be defined through direct measurements on site-specific samples (in the laboratory), or in situ measurements (e.g. pumping test). When this information is not available, *K* can be estimated from a hydrogeological knowledge of the region and published values of *K* measured in similar contexts for the same type of layers, defining a relation between the texture of sediments and *K* of superficial deposits (MacDonald et al. 2012). The available local information on shallow aquifers was limited. The data on existing pumping test found in the study area carried out in boreholes from the national database of DGPRE and SNAPE (Service Nationale de Points d’Eau, Guinée) indicate *K* values referred to deep fractured hard rock, while none of the pumping tests provide hydraulic parameters referred to unconsolidated shallow layers. Also, there are strong limitations on the availability of hydrogeological studies.

Different methods are available to estimate hydraulic conductivity from grain size of sediments. The following *K* values for different texture classes (as defined in Table 5) were obtained from literature (Domenico and Schwartz 1998; Fetter 1994; Freeze and Cherry 1979; Neuzil 1994; Sheperd 1989) and adopted:

- *K* = 10^−4^ m/s for coarse material (corresponding only to sand deposits in this region, as no gravel is present)
- *K* = 10^−5^ m/s for medium texture material
- *K* = 10^−6^ m/s for fine texture material

In the case of consolidated hard materials, *K* = 10^−6^ m/s was assumed (considering the presence of unconsolidated sediments filling the empty space of the hard layer). However, when hard rock represents more than 50% of the components, the layer is assumed to be the upper limit of the basement, and manual drilling cannot be performed. With the distribution of hydraulic conductivity lognormal, the standard practice was followed of calculating the weighted standard mean of values attributed to the individual lithology, both as percentages of each stratigraphic level and as components of the stratigraphic stretch analysed (Sanchez-Vila et al. 1995). In this way, the *K* value was estimated for each interval of 2 m, obtaining from the weighted average of log[*K*] of each texture class multiplied by its percentage, as shown in Eq.(2):

2 $$ \log {\left[K\right]}_{\mathrm{interval}}=\log {\left[K\right]}_{\mathrm{coarse}}\times \%{}_{\mathrm{coarse}}+\log {\left[K\right]}_{\mathrm{medium}}\times \%{}_{\mathrm{medium}}+\log {\left[K\right]}_{\mathrm{fine}}\times \%{}_{\mathrm{fine}}+\log {\left[K\right]}_{\mathrm{cons}.}\times \%{}_{\mathrm{cons}.} $$

where *K*_interval_ indicates the estimated hydraulic conductivity of the interval composed by a mix of different textural classes. At this point, it is possible to estimate the transmissivity in the exploitable layer (*T*_ex),_ multiplying the average *K* in the saturated layer (*K*_ex_, between the static water level and the upper limit of basement or the maximum possible depth of 50 m) and its thickness (*H*_ex_).

The output table was processed obtaining the following parameters for each borehole log: depth to hard rock, depth to water, thickness of hard lateritic layers, average estimated hydraulic conductivity and hydraulic transmissivity of exploitable layer. The meaning of “depth to rock” and “depth to water” was explained earlier. Since there is often no sharp transition from unconsolidated layers to hard rock, it was assumed that the depth to hard rock corresponds to the upper limit of layers having more than >50% of textural component in the output table classified as hard rock, while the depth to water was approximated with the data of static water level.

### Validation with measured *K* values from field tests

The estimated values of hydraulic parameters from the interpretation of stratigraphic logs were compared with measured parameters obtained from two field campaigns (May 2014 and March 2015) within the study area in Senegal involving pumping tests in large open wells, to obtain direct measurements of hydraulic parameters for the shallow aquifer (the expected target for manual drilling).

A total of 11 pumping tests were completed, covering different geological units. They are mainly distributed in the western and central part of the study area, as large-diameter wells are extremely rare in the eastern sector.

Since both field campaigns occurred in the late dry season, it was difficult to find wells with an adequate water column to carry out pumping tests for an extended period. Thus, the pumping phase was ideally undertaken for 1 h, but in several cases it was interrupted after a shorter period because the water column was too small to run the pump in a safe condition. The recovery phase was monitored for 1–1.5 h, which provided the most relevant information for the estimation of hydraulic parameters.

**Fig. 3:**
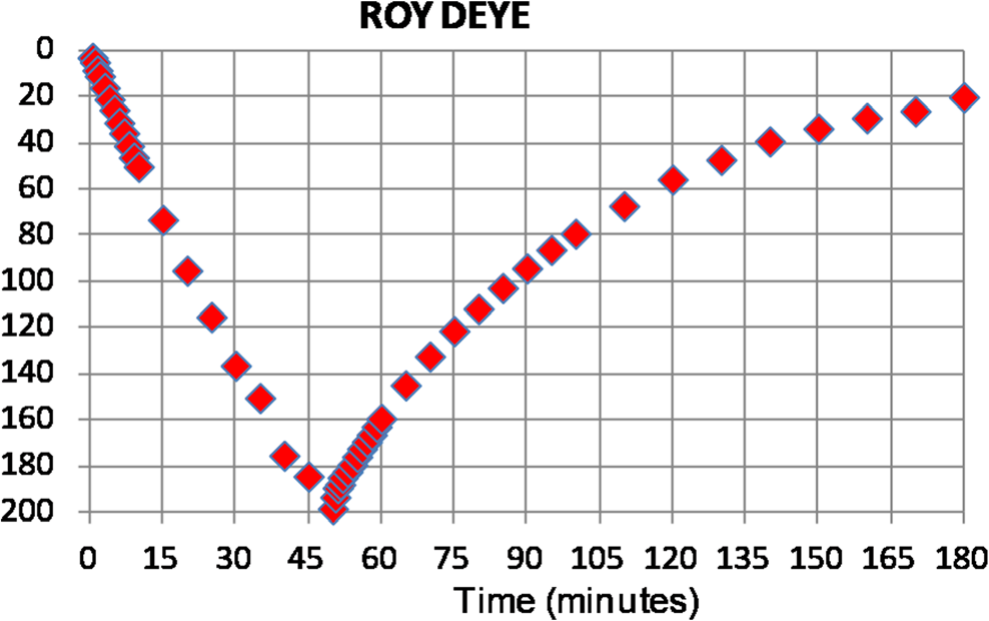
Example of a drawdown and recovery curve for a pumping test executed at Roy Deye (Louga region, Senegal)

Since the effect of storage capacity in large diameter wells is not negligible during the pumping phase, the recovery data are a better diagnostic of aquifer parameters than the drawdown, particularly for short periods of pumping (Barker and Herbert 1989). The methods to interpret recovery data based on the assumption of equilibrium between drawdown in the well and depression in the water table (e.g. Papadopulos and Cooper 1967; Herbert and Kitching 1981; Barker and Herbert 1989; Herbert et al. 1992) were not considered suitable. Observing the linear shape of the drawdown curve (Fig. 3) and the ratio between the volume of the well that was emptied during the test and the total volume of water extracted, a “slug test” approach for the interpretation was selected. Slug tests are good for the estimation of aquifer properties in hand-dug wells because they are commonly used in low-permeability environments, take into consideration the storage of water in the well, are easy to conduct in the field and are versatile (Mace 1999). They consider the conditions of an instantaneous water removal from the well and no contribution of water from the aquifer during the pumping phase.

Amongst the different methods proposed (e.g. Hvorslev 1951; Cooper et al. 1967; Bouwer and Rice 1976; Rupp et al. 2001; Uribe et al. 2014), Bouwer and Rice’s methods (and modifications by Rupp et al. 2001) seemed the most suitable for the interpretation, given the geometry of the system and the development of the test. In fact, this method was designed for the interpretation of slug tests in fully or partially penetrating wells, tapping unconfined aquifers.

**Fig. 4:**
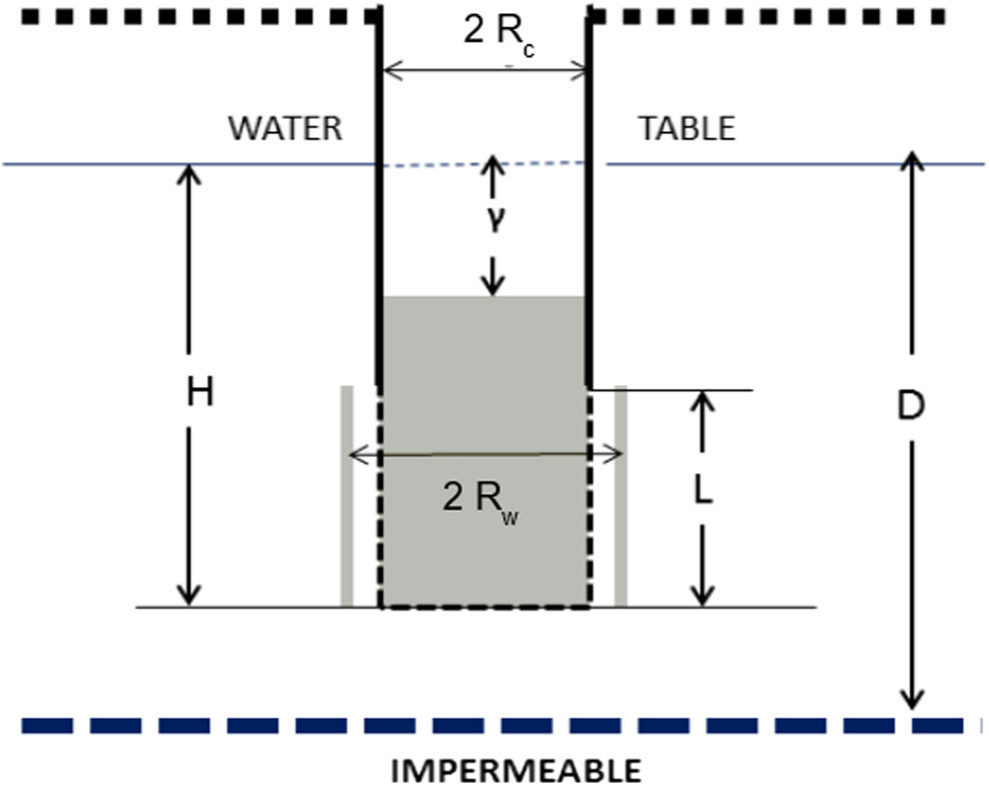
Geometry and symbols for partially penetrated wells in an unconfined aquifer. From Bouwer and Rice (1976). *H* = distance between water table and bottom of the well, *D* = distance between water table and bottom of the aquifer, *L* = length of screened section of the well, *Rw* = radius of the well, *Rc* = internal radius of concrete rings

*K* was estimated using Bouwer and Rice’s original method (Bouwer and Rice 1976) as well as the modified equation for ln (Re/Rw) proposed by Rupp et al. (2001) for soil classes Sa1 (Sand) and Lsa1 (Loamy sand) to take into account the influence of unsaturated hydraulic conductivity. Rw is the radius of the well, and Re is the effective radius over which the depression of static water level is dissipated (in Rupp’s method, this parameter depends on soil texture). The following assumptions were considered (Fig. 4):

1. Condition similar to fully penetrating wells (*D* = *L*). Considering that there is generally a concrete slab at the bottom of improved large-diameter wells in Senegal, water flow is therefore only horizontal and the base of the well can be considered an impermeable layer.
2. The well is fully screened, therefore filtration occurs along the whole well surface between the water table and the bottom of the well (*H* = *L*). Although the screened section of the well (perforated concrete rings) is smaller, the presence of gravel packing up to the water table facilitates filtration even from the shallower part of the aquifer.

## Results and discussion

### Comparison of *K* values obtained from analysis of stratigraphic logs and pumping tests

*K* values obtained from the set of pumping tests executed in large-diameter wells were compared with the estimation of hydraulic conductivity obtained from the interpretation of logs from boreholes located closer than 7 km from the pump-tested wells. This threshold distance was selected by calculating the minimum distance required to have at least two stratigraphic logs at an acceptable distance from the wells (i.e. a distance where the stratigraphic information of the borehole logs can be considered reasonably valid to represent the characteristics of shallow geological layers at the well’s position). The radius of 7 km can lead to a discrepancy in geological conditions between the open well and surrounding stratigraphic logs, especially in the presence of lateral variations (generally limited in the study area) and undulated topography. However, the low density of borehole logs (i.e. the large distance between stratigraphic data) made it difficult to assume a smaller threshold value and the researchers were obliged to accept this source of error in the comparison of *K* values. The interpretation considered the texture of those geological layers at the same depth as the saturated interval in the well. As observed in Table 7, the mean and median of the difference in the estimation of *K* obtained through the interpretation of pumping tests (direct measurement in the field) and processing of borehole logs is limited. This difference has been estimated for only seven pumping tests (out of the 11 that were executed), as some of the tests gave results considered not reliable because of the limited water column in the wells and difficulty in properly measuring the change in water level.

**Table 7:** Estimation of mean and median of the difference between Log *K* obtained through interpretation of pumping tests and borehole logs (7 samples). *K* expressed in m/s

Statistic	*K* Rupp’s method (assuming soil class Sa1)	*K* Rupp’s method (assuming soil class Lsa1)	*K* Original Bouwer and Rice method
Mean	0.42	0.44	1.22
Median	0.25	0.28	0.48

The results providing the best fitting with *K* value obtained from stratigraphic logs (giving the lowest mean and median value of the difference) were obtained using Rupp’s method and considering sandy soil; in 5 out of 7 cases the difference in Log *K* between the two methods is smaller than 0.5. Two points gave a higher value of difference in *K* value; one possible justification can be the presence of variations in texture of shallow geological layers between the open well (where the pumping test was carried out) and the surrounding borehole.

Therefore, it can be considered that the *K* estimated during the analysis of stratigraphic logs by using TANGAFRIC is reasonably consistent with the results of pumping tests, and is possibly slightly underestimated. One also has to consider that this method (comparing *K* values from pumping tests in large wells with the surrounding borehole logs using texture and K data of layers at the same depth as the static water level of the pumping test) is based on the assumption that there is no lateral modification of texture and *K* (only vertical modification) in the area and that topography is flat; if these conditions are not taken into account, they can introduce other relevant factors that may make this comparison less correct. On the basis of geological and morphological features of the study area, it was considered reasonable to assume limited lateral variability and thus confirm the validity of the method.

### Characterizing the shallow aquifer and the suitability for manual drilling based on borehole-log positions

The method for the assessment of suitability for manual drilling was applied to the study area, and the feasibility, potential for exploitation and suitability were elaborated using maps. The results of the semi-automated analysis of borehole logs (and the comparison with direct field measurements) show the distribution of the different hydrogeological parameters considered and assign the appropriate class of suitability at borehole-log positions. The distribution of the different parameters to assess suitability in the study area is discussed.

#### Classification of feasibility for manual drilling

##### Depth to hard rock

**Fig. 5:**
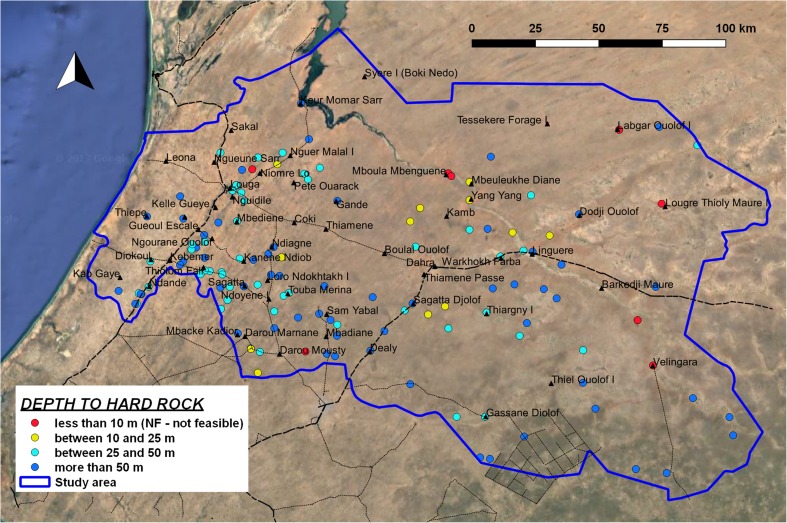
Map of depth to hard rock, extracted from borehole logs

In most of the study area, the bedrock (i.e. the upper limit of tertiary sedimentary rocks under the unconsolidated deposits) is deeper than 10 m, therefore satisfying the criteria defined in the feasibility model for manual drilling (class F and class FS). In 52% of the cases, bedrock is deeper than 50 m, therefore manual drilling can reach its maximum depth without limitation because of geological conditions. The unconsolidated shallow layer presents a small thickness in many of the boreholes concentrated in the eastern sector, although there is high variability in the same area (Fig. 5). As a consequence, there are limitations associated with the exploitable water column in hand-drilled wells.

##### Depth to water

**Fig. 6:**
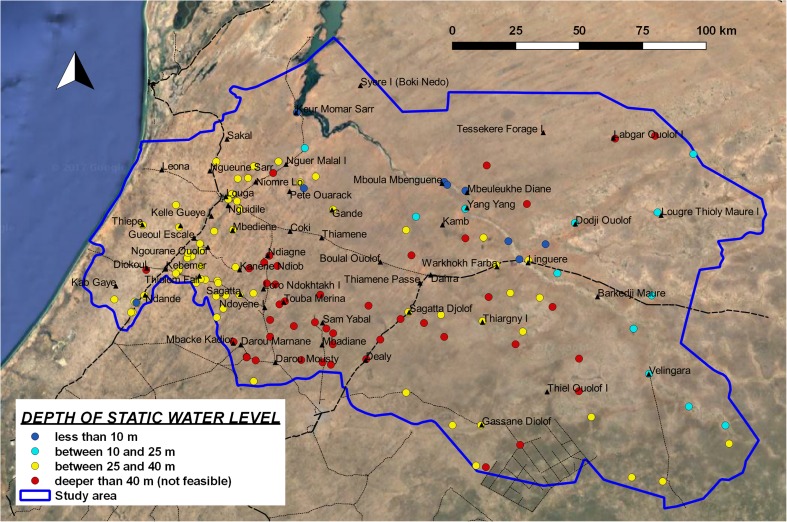
Map of depth to static water level, at borehole-log positions

This is the most important limiting factor for the implementation of manual drilling in the study area. 45% of the logs show a static water level deeper than 40 m (Fig. 6); therefore, in this situation, manual drilling is considered NF according to the limits defined in the proposed classification model (Fig. 2, condition 2). The average value of water depth is 35 m, with a standard deviation of 10.3 m. However, estimating the depth of exploitable water from information on static water level of deep boreholes can lead to an unreliable interpretation in the case of boreholes that tap confined deep aquifers. In this situation, hand-drilled wells can only reach exploitable water that is much deeper than the estimated water level, and in some cases this may be not feasible. This situation is frequent in the central and eastern sector of the study area, where the true depth of the shallow water table can be estimated from data on static water levels in large-diameter wells. Since this information is rarely registered in the national database, direct field observation is suggested.

The most critical zones are located SE of Kebemer, in the SW sector of the study area, where the depth of groundwater makes manual drilling not feasible in several locations. Further east, there are more sites where the water depth is shallower than 25 m. However, two relevant aspects must be considered in this zone: the limitation in drilling depth because of the presence of hard rock can make it difficult to have a sufficient water column in the well. Also, the extreme scarcity of large-diameter wells can indicate the presence of confining layers and possible errors in the estimation of the depth of the shallow water table. The coastal strip has no borehole log information, but the direct observation of large-diameter wells in the field indicates that groundwater is shallow (in most of the cases not deeper than 10 m).

##### Thickness of hard laterite

**Fig. 7:**
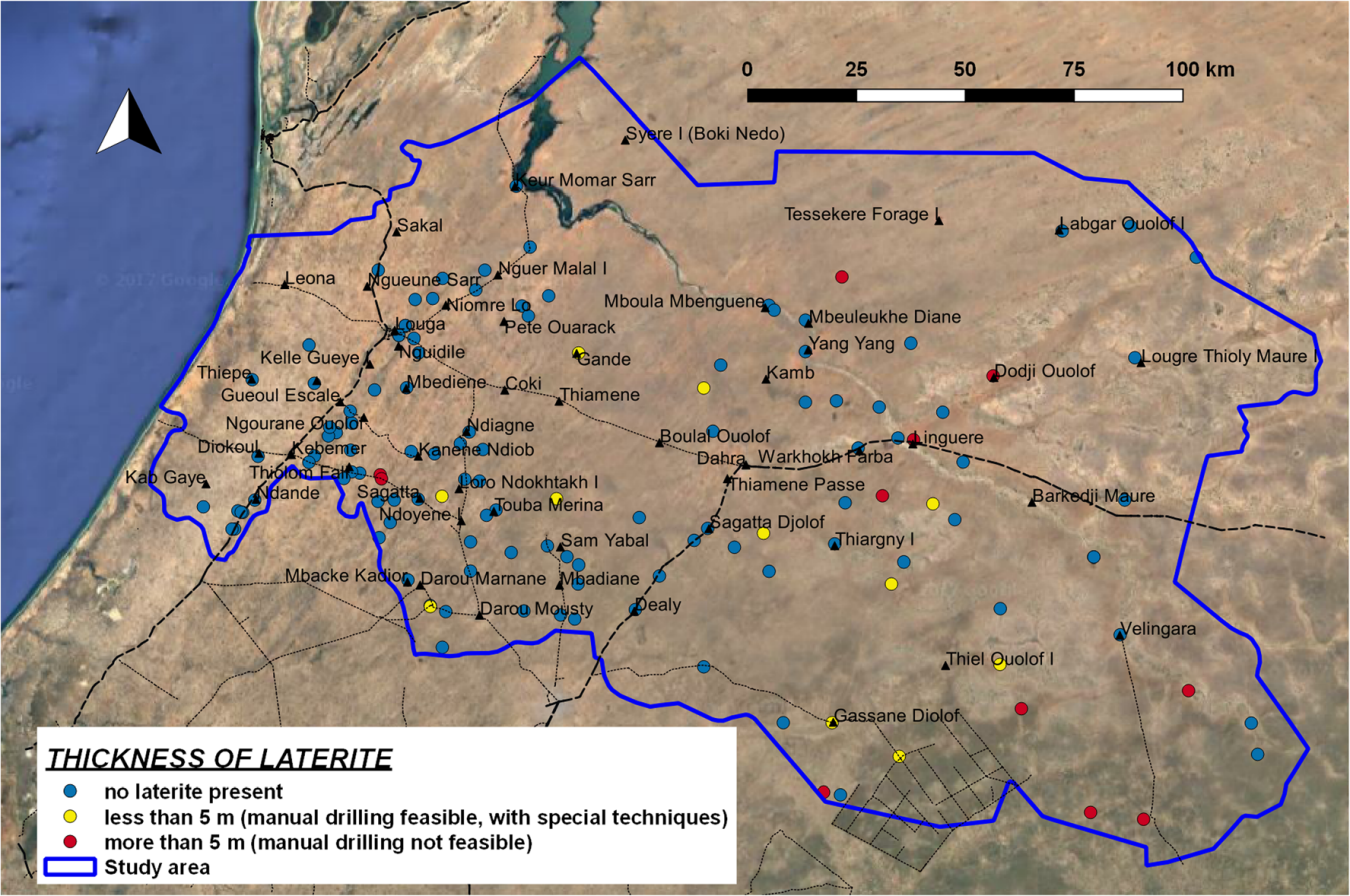
Thickness of laterite layers in stratigraphic logs

Hard laterite is not frequently found in the study area (Fig. 7). Over much of the area there is generally no laterite; therefore, it does not represent a limiting factor for the implementation of manual drilling (Fig. 2, condition 3). The presence of thick hard lateritic layers can represent an obstacle to feasibility (when laterite is thicker than 5 m the condition is considered not feasible, class NF) only in the south-eastern side of the study area, and in some locations in the centre (around Linguere). Furthermore, in the southern part of the region there are a number of logs showing the presence of laterite with thickness less than 5 m; in this situation, manual drilling is considered feasible, but percussion techniques are required (class F-SP).

##### Assessment of feasibility for manual drilling

**Fig. 8:**
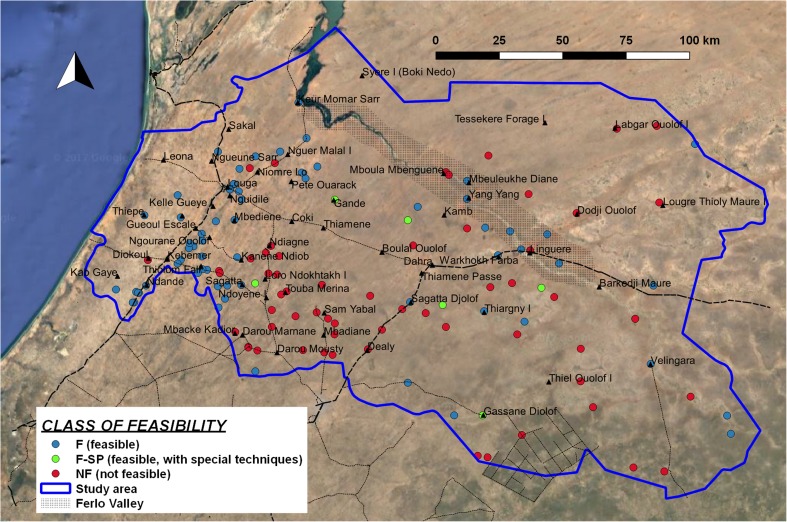
Classes of feasibility for manual drilling, at borehole-log positions

After having calculated the three key parameters (depth to hard rock, depth to water, thickness of laterite), it is now possible to assign a specific class of feasibility for each of the borehole logs (Fig. 8), following the procedure explained in Fig. 2. In the whole study area, 62% of the borehole logs show feasible conditions for manual drilling. Along the western coastal strip there are feasible conditions in almost the whole group of logs, while in the Ferlo Valley the trend is mixed, with a predominance of feasible conditions. Limitations in the feasibility of manual drilling are present in the southern part (mainly because of the depth of the water level) and the NE sector (in this case, the main constraint is the limited thickness of unconsolidated layers).

It is important to underline that the presence of confined aquifers and the lack of direct data on water levels in large wells can lead to unreliable information concerning the depth of the shallow water table in the eastern part of the study area and impact the estimation of feasibility of manual drilling.

#### Classification of suitability for manual drilling

##### Hydraulic conductivity of exploitable saturated layer

**Fig. 9:**
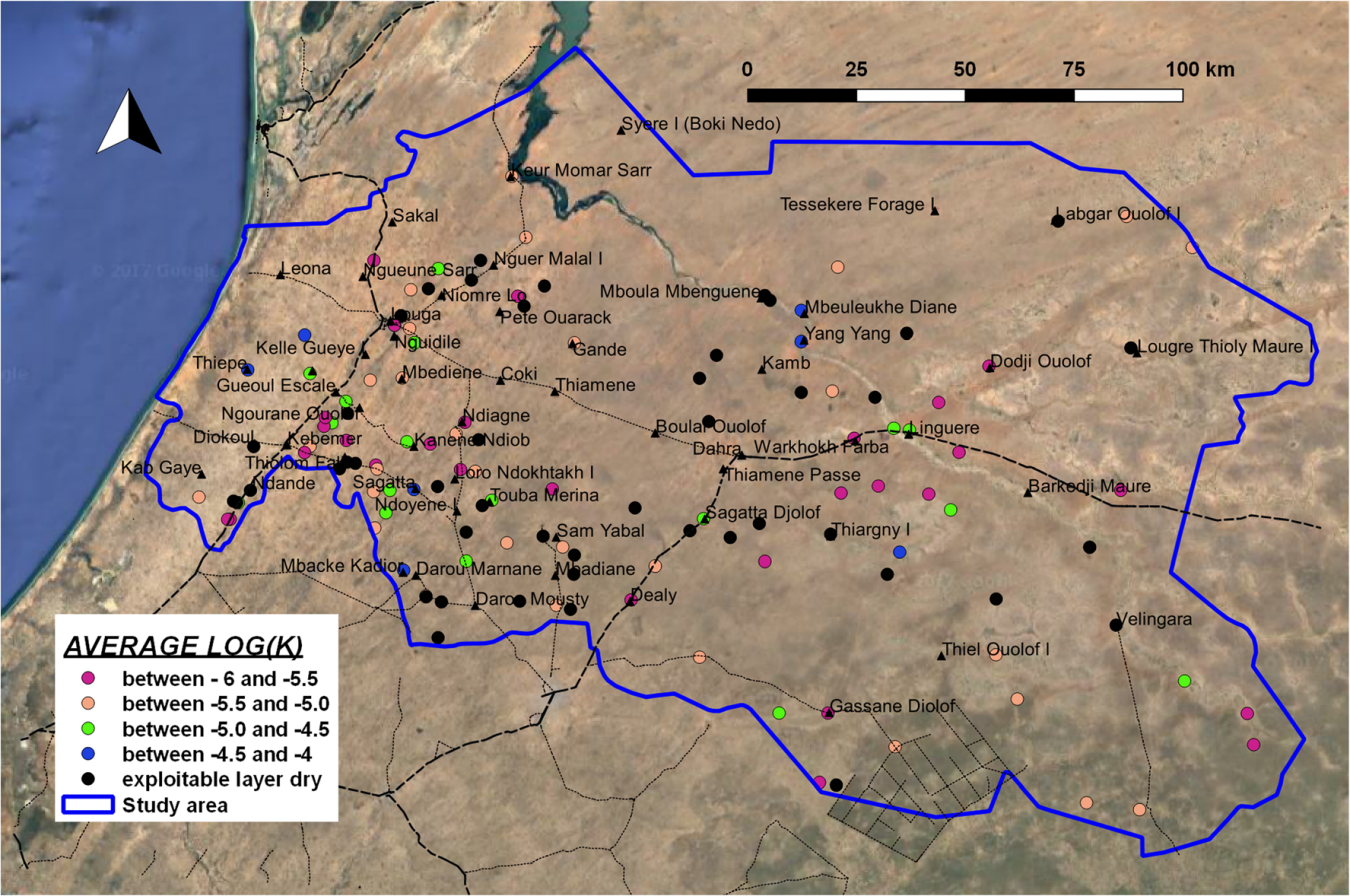
Map of average Log(*K*) in the saturated layer up to 50 m (hydraulic conductivity, *K* in m/s)

The estimated hydraulic conductivity of the porous shallow aquifer in the exploitable layer shows Log(*K*) values characteristic of an intermediate texture class; considering that the medium grain size fraction is limited, the *K* value can be attributed to a mix of sandy deposits with an important clay fraction. Average Log(*K*) is −4.83 (corresponding to *K* = 1.5 × 10^−5^ m/s), with a standard deviation of 0.4. The lowest *K* values in the exploitable layer are found SE of Kebemer and in the eastern sector of the study area. The highest values are in the coastal zone. Furthermore, a few boreholes show a high estimated *K* value in the exploitable layer along the river valley NW of Linguere (Fig. 9). In general, hydraulic conductivity in the first metres is higher than in the deeper part of the porous aquifer: the average value of Log (*K*) between 0 and 10 m deep is −4.4 (*K* = 4.10 × 10^−5^ m/s), while between 20 and 30 m, it is −5.0 (*K* = 1.10 × 10^−5^ m/s); in both cases standard deviation is 0.4 or 0.5. This is related to a general trend (90% of the whole data set) of decreasing coarse textural fraction in the deeper layers of the shallow porous aquifer.

##### Thickness of exploitable saturated layer

**Fig. 10:**
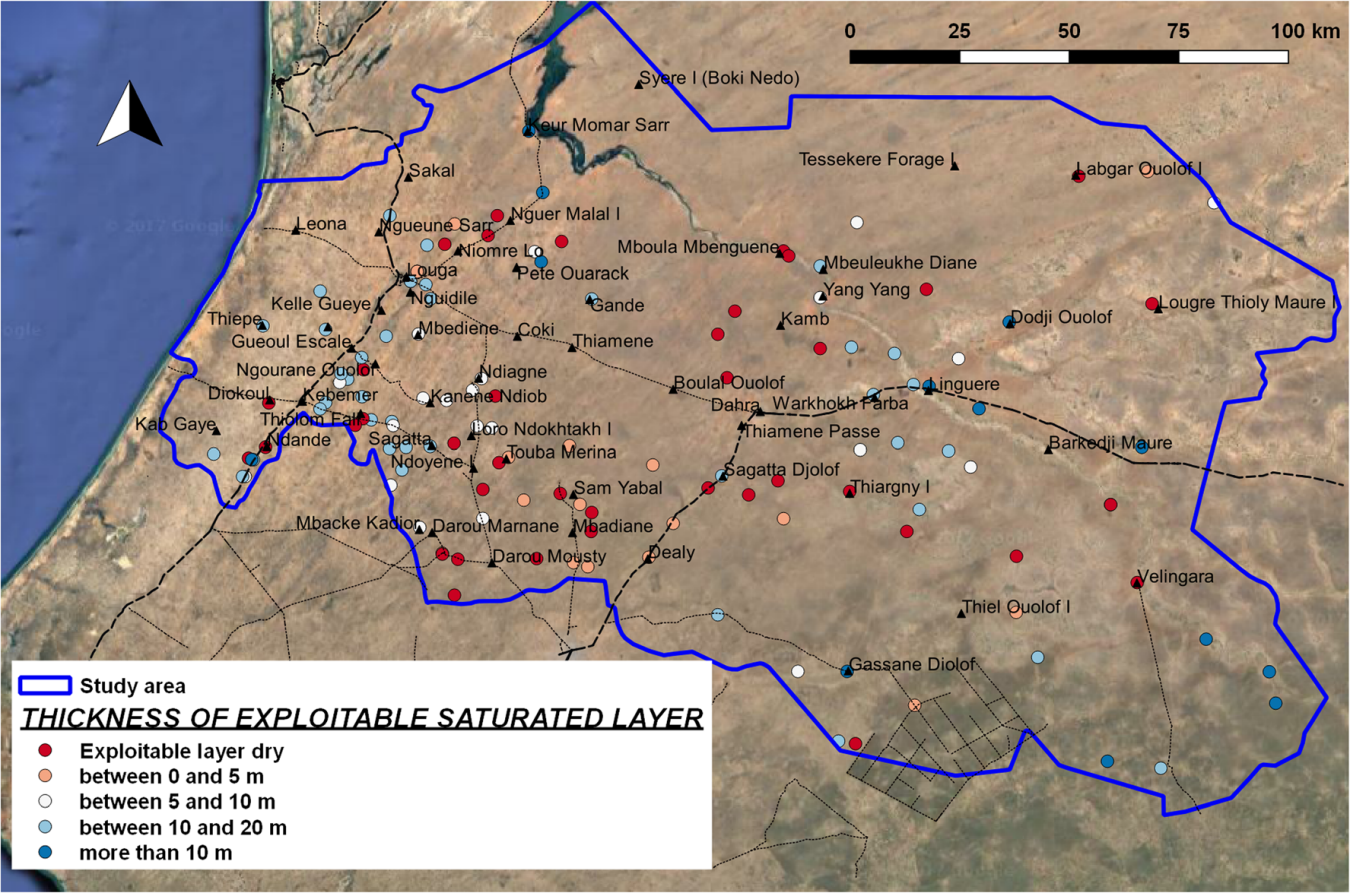
Map of thickness of exploitable saturated layer, at borehole-log positions

Comparing the depth to hard rock and the depth to water, the thickness of the saturated layer is obtained. For the classification of suitability for manual drilling, only the layer at maximum depth of 50 m is considered exploitable. Furthermore, manual drilling is not considered feasible when the exploitable layer is thinner than 10 m. Thirty percent of the 131 boreholes do not have an exploitable saturated layer, as the water level is deeper than the depth to hard rock (or deeper than 50 m), while another 29% of logs show an exploitable layer thinner than 10 m; therefore, they are not considered to have suitable conditions for manual drilling. Forty-one percent of boreholes have sufficient thickness of saturated layer for the implementation of manual drilling, although in reality the possible effect of confining layers in the eastern part can lead to a different (and less favourable) situation (Fig. 10).

##### Potential for exploitation with manual drilling

**Fig. 11:**
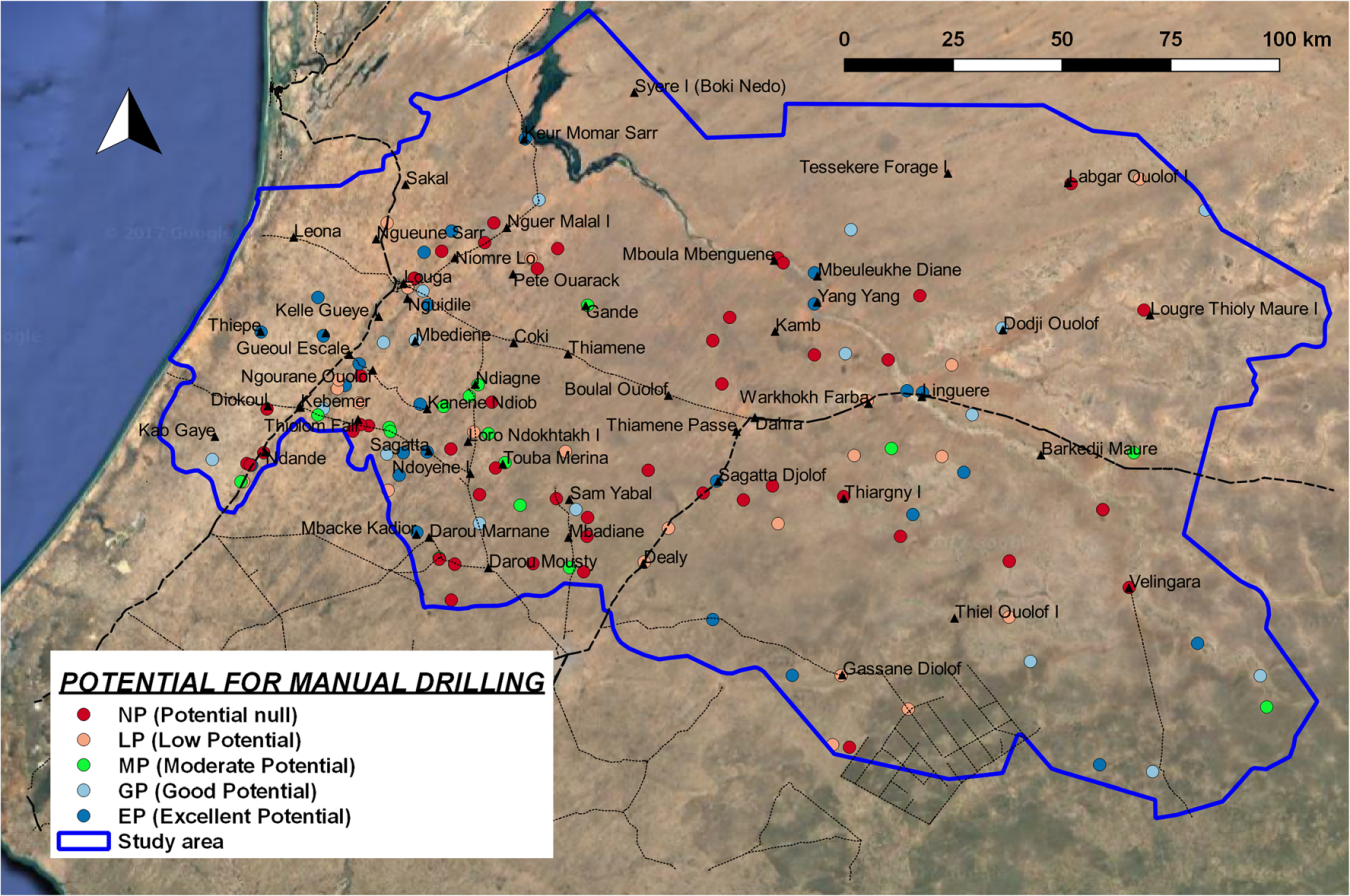
Map of potential for manual drilling, based on *T*_ex_. *NP* potential null, *LP* low potential, *MP* moderate potential, *GP* good potential, *EP* excellent potential

The threshold values of *T*_ex_ that discriminate the different classes of potential (Table 3) on the basis of site-specific conditions are assigned using an approximate relation between *T*_ex_ and expected drawdown with a hand pump (expected yield 0.2 L/s) and solar pump (expected yield 1 L/s) using Dupuit’s equation for steady flow. The drawdown is compared with the site-specific condition of the expected maximum water column in the well (depending on the depth of groundwater) and seasonal fluctuations. In this way, it is possible to estimate the sustainability of a continuous and intense pumping. Considering the hydrogeological context of the study area, the threshold values of *T*_ex_ shown in Table 8 were assumed. With this classification, it is possible to assess the distribution of potential in the whole study area (Fig. 11).

**Table 8:** *T*_*ex*_ limits of the five class of potential for manual drilling

Class of potential	Range of *T*_ex_ (m^2^/s)
NP: potential null	0
LP: low potential	Between 0 and 3.10 × 10^−5^
MP: moderate potential	Between 3.10 × 10^−5^ and 5.10 × 10^−5^
GP: good potential	Between 5.10 × 10^−5^ and 1.3 × 10^−3^
EP: excellent potential	More than 1.3 × 10^−3^

##### Suitability for manual drilling

**Fig. 12:**
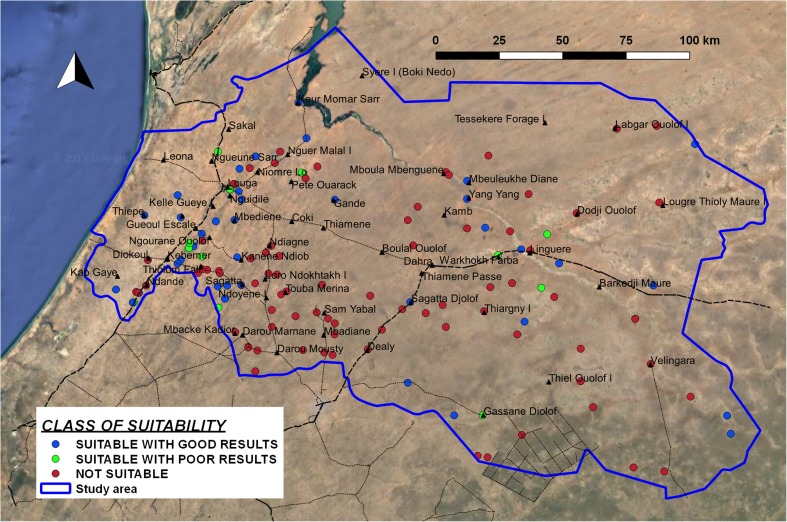
Classification of suitability for manual drilling at borehole-log positions

On the basis of the different hydrogeological parameters described previously, it has been possible to classify each borehole log in terms of feasibility and potential for manual drilling. The final class of suitability for manual drilling can be obtained from the combination of these two, as described in Table 4. The results of classification of suitability at borehole-log positions are shown in Fig. 12.

In those areas where manual drilling is considered feasible, one can observe a good potential in the coastal area and in the Ferlo valley (i.e. along the dry river between Mboula and Linguere, in the centre of the study area). However, in this zone, the assessment of suitability can be not reliable, given the uncertainty about the real depth to water level in the shallow porous aquifer and the difference between direct field observations in large-diameter wells and static water level data from deep boreholes in the national database, as previously explained. On the other hand, the area eastward from Kebemer (i.e. the region of Ndiob, Ndoyenne, Darou, in the SW sector of the study area) has several places with low potential, because of the double effect of the depth to water (therefore a small water column in the well, if maximum depth is 50 m) and a higher percentage of fine materials (with a low value of *K*). In the case of drilling deeper than 50 m, both estimated feasibility and potential would improve in this region, while in the NE sector the limitations imposed by the presence of shallow hard rock would not change the estimation of suitability. The estimation of feasibility depends on the limits of depth defined for manual drilling based on the experience of drillers. In this research, a maximum well depth of the wells at 50 m (and a maximum depth to exploitable water at 40 m) was assumed. However, 20% of the data shows a water depth between 40 and 50 m. The class of feasibility for these points would change in the case of increasing the maximum estimated depth of drilling by 10 m (this would be considered feasible).

## Conclusion

This research allowed for the delineation of suitable zones to implement manual drilling in a region of northwestern Senegal, improving (i.e. increasing the scale of detail and the reliability of the interpretation by validation with real stratigraphic and hydraulic data) the previous map of suitable zones in this country (Kane et al. 2013).

During the research, a specific software (TANGAFRIC) and a structured method to carry out a semi-automated analysis of stratigraphic information through organization, codification and processing of existing borehole-log data have been developed. This activity was completed on a sample of 173 borehole logs (through manual input of stratigraphic codes carried out by technical staff in Senegal, followed by an automated procedure of data elaboration) in Louga region, but the process can be replicated with other data sets. In Senegal, the national database contains a detailed description of 1,419 stratigraphic logs scattered throughout the whole country, but many more are available in hard copy at the regional branches of the national water authority. The update of information concerning existing water points is still a difficult aspect; in recent years, increasing attention has been given to setting up an efficient system for water point mapping and monitoring. The lack of up-to-date information strongly limits the reliability of studies on the functionality of water supply and access to safe water, while it is less relevant for hydrogeological interpretation. The information stored in the national database is generally collected at the moment of construction of water points, but most of the hydrogeological data (especially stratigraphic description) does not change over time and the information is still correct.

In other countries in Africa, large databases of water points are stored by the national institutions, with limited capacity to analyse this information and exploit it to formulate hydrogeological interpretation. Tools and methods proposed in this research could provide a valid support to study not only the shallow hydrogeology but also deeper fractured formations, contributing to groundwater exploration and management.

In the next few years, manual drilling is expected to spread in several countries. Various international organizations and funding agencies are committed to supporting this process—for example, UNICEF and its partners PRACTICA Foundation and RWSN (Rural Water Supply Network) are running an important technical assistance program to create a highly professional manual drilling sector in many countries in Western and Central Africa (Central African Republic, Togo, Mauritania, Mali, Ivory Coast, Guinea, Niger, Nigeria, DRC) funded by DGIS (Directorate-General for International Cooperation of Dutch Government), DFID (Department of International Development of UK Government) and other donors. It would be important for scientific research to provide valid tools for effective planning and implementation of this process, which could produce a highly positive impact on access to safe water and the living conditions of the population.
